# Commercial techniques for preserving date palm (*Phoenix dactylifera*) fruit quality and safety: A review

**DOI:** 10.1016/j.sjbs.2021.04.035

**Published:** 2021-04-20

**Authors:** Mohammad Sarraf, Monia Jemni, Ibrahim Kahramanoğlu, Francisco Artés, Shirin Shahkoomahally, Ahmad Namsi, Muhammad Ihtisham, Marian Brestic, Mostafa Mohammadi, Anshu Rastogi

**Affiliations:** a Department of Horticulture Science, Shiraz Branch, Islamic Azad University, Shiraz 71987-74731, Iran; b Regional Research Center in Oasis Agriculture of Degache, Tunisia; c European University of Lefke, Faculty of Agricultural Sciences and Technologies, Gemikonagi, via Mersin 10, 99780 Northern Cyprus, Turkey; d Postharvest and Refrigeration Group, Department of Food Engineering, Universidad Politécnica de Cartagena (UPCT), Paseo Alfonso XIII, Cartagena, Murcia, Spain; e Institute of Plant Biotechnology, UPCT, Campus Muralla del Mar, Cartagena, Murcia, Spain; f Department of Horticultural Science, University of Florida, Gainesville, FL 32611, USA; g College of Landscape Architecture, Sichuan Agricultural University, Chengdu, Sichuan 611130, China; h Department of Plant Physiology, Slovak University of Agriculture, A. Hlinku 2, 949 76 Nitra, Slovakia; i Young Researchers and Elite Club, Bushehr Branch, Islamic Azad University, Bushehr, Iran; j Laboratory of Bioclimatology, Department of Ecology and Environmental Protection, Poznan University of Life Sciences, Piątkowska 94, 60-649 Poznan, Poland

**Keywords:** *Phoenix dactylifera*, Dates, Handling, Storage, Sustainable treatments

## Abstract

The popularity of date palm (*Phoenix dactylifera*) fruit is increasing, therefore the demand for high-quality date palm fruit with less or no chemical treatment is the topic of interest for date producers and consumers. The quality of date palm fruit is much dependent on its postharvest handling and processing. For preventing the degradation and maintenance of the high quality of dates during the storage an appropriate harvest and post-harvest processes are required. The process should control the biotic and abiotic factors like insects, fungus, temperature, as well as handling and processing of dates. Therefore, in this work, we reviewed the literature related to the protection of date fruits during their post-harvest life. The commercially viable advance and updated techniques that can be used to avoid storage losses and problems while keeping fruit quality (nutritional, color, flavor, and texture) and microbial safety under optimal conditions are discussed.

## 1 Introduction

Date palm (*Phoenix dactylifera*) is probably the oldest tree cultivated by humans. Historical evidence has shown that their fruits were cultivated and used for thousands of years (Popenoe, 1913, Tavakolian et al., 2013). It seems to have a 7,000-year history that coincides with the first human civilizations from northeastern Africa to the northwestern Tigris and Euphrates rivers (Ashraf and Hamidi-Esfahani, 2011, Dawson, 1982). For people in the Middle East and North Africa, dates are more than just a fruit. The date palm is a tree that was mentioned in Islam, Christianity, and Judaism and it used to be the food of most people (Ashraf and Hamidi-Esfahani, 2011, Munier, 1973). It is the most important way of nutrition and livelihood in most arid and semi-desert areas (Awad, 2007, Botes and Zaid, 1999). Countless sources have examined the history of date cultivation and domestication. In ancient Greece, this plant was called Phoenix because of its transmission and cultivation by the Phoenicians in the Mediterranean region at that time (Ashraf and Hamidi-Esfahani, 2011). Due to its high carbohydrate and other nutrients content date fruits have been used as a complete and nutritious food in military trips and other caravans throughout history. After eating the fruit, they inadvertently dispersed the plant's seeds in various areas around the word (Ashraf and Hamidi-Esfahani, 2011, Barreveld, 1993).

Dates are known for their numerous food and industrial uses (Munier, 1973, Shaghaghian et al., 2014). Thousands of years ago, humans realized their importance, and the inclusion of dates in the diet is currently associated with several positive effects on human physiology (Hussain et al., 2020, Khalid et al., 2017). Its fruits are known to include an abundant amount of essential nutrients and are considered as a complete food with carbohydrates, fiber, and lipids present in significant amounts. In addition, dates are shown to have a high antioxidant capacity (Biglari et al., 2008, Hussain et al., 2020) and diabetes-reducing properties (Mard et al., 2010, Salehi et al., 2019). All of these shows that date fruits are very important for a healthy diet and they contribute to the improvement of human health.

Thousands of date cultivars exist around the world (Chao and Krueger, 2007). It can grow in hot and low humidity areas under any type of soil and is known to be tolerant to saline conditions as compared with many other cultivated plants (Lobo et al., 2014). The growth rate and development of date fruit follow a sigmoid growth curve (Chao and Krueger, 2007). The growth of date palm and its shelf life is known to be determined by different environmental factors (including precipitation, birds, wind, insects, microbial attacks, temperature, humidity, etc.).

It has been shown that the nuclear size of a date palm genome is about 670 Mb which is distributed among 18 chromosomes. A diploid date palm has 2n = 2x = 36 of chromosome number per pair (Al-Mssallem et al., 2013). Improved genomic technology and better research based on phenotype characteristics in population genomics provides an opportunity for the researchers to investigate the origin of the date palm tree (Al-Mssallem et al., 2013). There are huge differences between cultivated and wild date palms from morphological and genetic perspectives, and their natural distribution is unidentified (Gros-Balthazard et al., 2018).

**Fig. 1:**
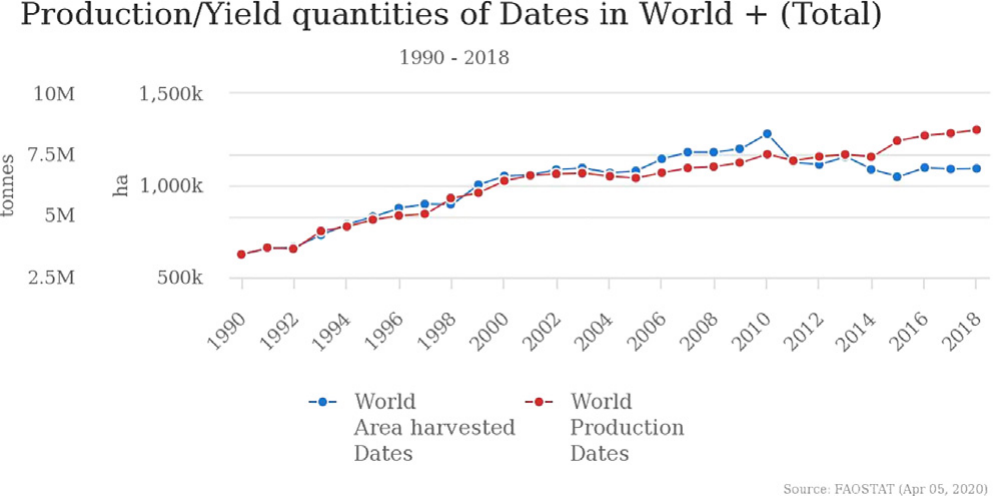
World Production of Date Palm “between” 1990–2018 (FAOSTAT, 2018).

The total production of dates fruit was recorded to be 3.43 million tons in 1990 which was harvested on about 625,000 ha area. Over the past three decades, the demand for global markets has increased reaching 8.52 million tons in 1.092,000 ha, Fig. 1. Egypt, Iran, and Saudi Arabia have always been the largest producers of date palm, and a large share of their exports to these countries is allocated (FAOSTAT, 2018).

**Fig. 2:**
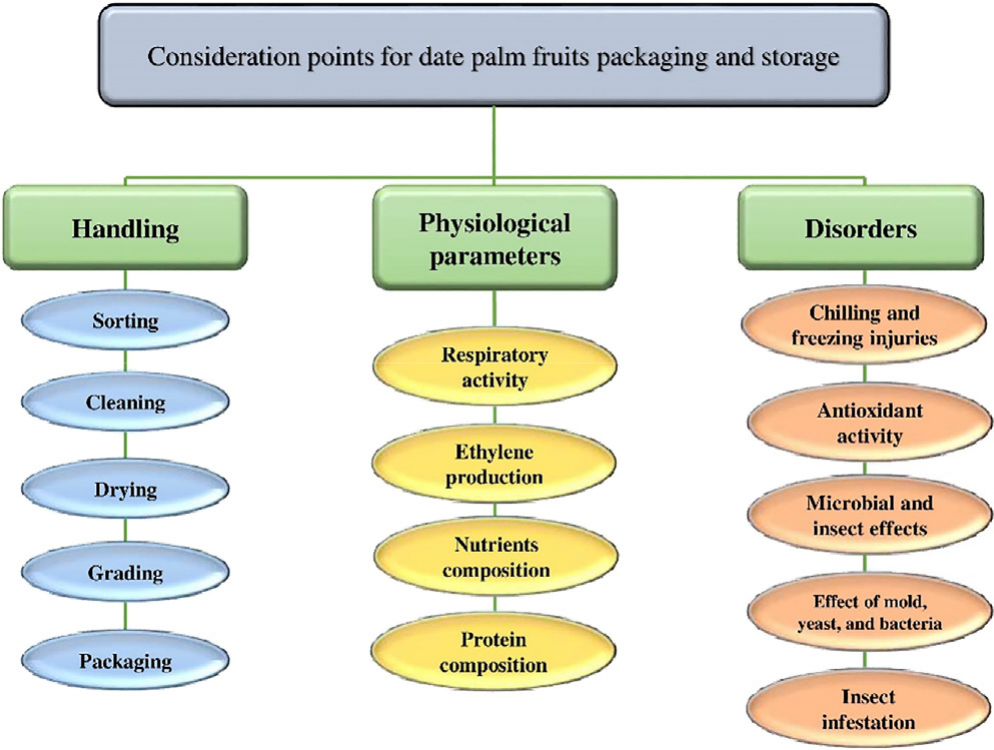
A systematic representation of consideration points for date palm fruits packaging and storage.

Due to the importance and growing acceptance of date fruits, it is a need of time to discuss the advantages and disadvantages of past and current fruit processing techniques. Therefore, in this review article, we have discussed past and modern processes to process and store date fruits for their better and profitable consumption. Fig. 2 shows the important consideration factors for the handling and storage of date fruits which makes the trade of date profitable for its producer and consumers and described in the following section.

## 2 Handling, treatments, and storage conditions required for dates

**Fig. 3:**
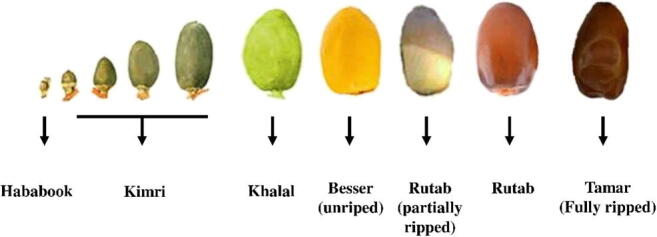
Stages of maturation of date palm fruits.

The date fruit can be classified into four categories: Fresh (used as fresh, Barhee variety), Wet (maturation achieved by storing at low-temperature or refrigeration, Hayany variety), Semi-dry (Deglet Noor and Medjool varieties), and Dry (Ameri, Halawi, Khadrawy, Thoory, and Zahidi varieties). Further, the date fruits are consumed, stored, and distributed according to their moisture content such as sweet Khalaal (yellow or red with 50–85% moisture content), the Rutab (light brown with 30–45% moisture content), and the Tamar (amber to dark brown with the moisture content of <25%) (Navarro, 2006). Fruits in the Tamar stage are resistant to microorganism infection. In Fig. 3, we showed the six stages of date palm ripening from Hababook to the Tamar stage. In table 1 we have also presented different methods to preserve the date food from different degrading agents as mentioned below, whereas table 2 presents some of the reported work where different biological, physical or chemical factors are mentioned to cause a negative impact on date fruits.

**Table 1:** Different preservation methods for date palm fruit.

Methods/Techniques employed	Advantages	Disadvantages	Reference
Refrigeration	Slow down enzymatic reactions, as well as the activity of microbial and insect’s life	The efficacy of low temperatures depends on (i) the inherent initial quality attributes of the dates, (ii) the microbe and insects present at harvest, and (iii) the temperature applied. Cooling does not prevent insect infestation although it reduces it, however, insect infestation does not take place at temperature below 4 °C, but at these levels the insects will not necessarily be destroyed	(Lallouche et al., 2017)
Fumigation	Killing insect life in all its stages of development: egg, larva, pupa and adult	Treatment must not be applied to fresh fruits or when stored under deep refrigeration, and the average practical dose is 15 g/m^3^ for 12–24 h at 15–16 °C Fumigant is slow acting Does not stop insects to emigrate from the dates (disinfestation) and insects in various countries have developed resistance to this gas The residue levels after treatment stay within the MRL (maximum residual levels)	(El-Mohandes, 2010, Navarro, 2006)
Heat Treatment	100% mortality of emigration and control of nitidulid beetles in dates	Cause discolouration and have a blistering effect that separates the skin from the flesh of the fruit	(Finkelman et al., 2006, Kader, 2009, Navarro, 2006, Rafaeli et al., 2006)
Modified Atmosphere Packaging (MAP)	The most effective to preserve the quality maintenance of dates with limited effects on the appearance of physiological disorder signs	Limited effects on the appearance of date fruits	(Al-Redhaiman, 2005, Dehghan-shoar et al., 2010, Kader, 2009)
Edible Coating	Improve the appearance of dates, protect the fruits, and reduce stickiness in case of soft dates Reduce water evaporation from the fruit and thereby reduce its weight loss by up to 50% and maintain the quality of the fruits	Some negative influence mainly on sensory attributes and flavor	(Aboryia and Omar, 2020)
Ozone(O_3_) Treatment	Reduce or eliminate all life stages (adults, larvae, and eggs) of Indian meal moth (*Plodia interpunctella*) and sawtooth grain beetle (*Oryzaephilus surinamensis*) The O_3_ is a generally recognized as safe sanitizer in the food industry It is a strong oxidizing agent, very effective on microorganisms Enhance total phenolics contents	Effectiveness depends on the application dose, temperature, duration, pH and soluble solids in water and even on the method of washing, as dips or drench. Bacteria are more sensitive to O_3_ than yeasts and fungi, Gram-positive bacteria are more sensitive to O_3_ than Gram-negative organisms and spores are more resistant than vegetative cells. O_3_ induced changes in surface color of some treated products, and their antimicrobial effect during storagewas variable depending on the type of microorganism, characteristics of fresh produce and prevailing storage conditions.	(Jemni et al., 2014a, Jemni et al., 2014b, Niakousari et al., 2010)
Electron-Beams	100% for all electron-beam treatments, also hatches were less compared to microwave, steaming and fumigation	The high cost as known with irradiation and other electromagnetic rays, and technical feasibility	(Al-Farsi et al., 2010)
The UV-C light	The main advantages of UV-C light is that it does not leave any residue, is lethal to most types of microorganisms, is easy to use without extensive safety equipment, and does not have legal restrictions	Exposure of cells to visible light after UV-C treatment induces enzymatic photorepair and expression of excision-repair genes that may restore DNA integrity	(Jemni et al., 2014a)
Electrolyzed water	Electrolyzed water involves on-site production of the disinfectant, which means there are no chemicals to store or handling costs for dealing with them Electrolyzed water is not corrosive to skin, mucous membranes, or organic material, hile having a high oxidation reduction potential with strong effect against microorganism	The effects of Electrolyzed water on by-product formation should be more studied	(Jemni et al., 2014a)

**Table 2:** Biological, physical, and chemical factors reported to cause a deteriorating impact on date palm fruits.

Factors	Alterations cause	Effect on dates	References
Microbiological	Fungal species: *Aspergillus* spp., *Alernaria* spp., *Stemphylium botryosum*, *Cladosporium* spp., *Macrosporium* spp., *Citromyces ramosus*, *Phomopsis diospyri*, etc	Attack fruit in the khalal stage and the Rutab stage. Growth of Aspergillus flavus on dates can result in aflatoxin contamination that would make them unsafe for human consumption and unmarketable	(Warner et al., 1990) (Yahia et al., 2014)
	yeast genera: *Saccharomyces*, *Hanseniospora* and *Candida* spp, *Zygosaccharomyces*	Yeast-infected dates develop an alcoholic odor (become fermented). Fermentation by yeast also results in souring of dates due to accumulation of ethanol and/or acetic acid with moisture content above 25% when kept at temperatures above 20 °C and its severity increases with duration and temperature of storage. Storage at low temperatures reduces incidence and severity of souring	(Warner et al., 1990, Yahia et al., 2014)
	Bacteria: *Escherichia coli, Staphylococcus aureus* and *Bacillus cereus, Acetobacter*	Bacteria are responsible for the acidification of dates (transformation of sugar in lactic acid or citric acid) (Warner et al., 1990). Lactic acid bacteria are present only at the Rutab stage in some varieties	(Warner et al., 1990, Yahia et al., 2014)
Physical	The physical alterations, commonly of mechanical origin, occurs during different operations of date’s manipulation (shocks, crushing and drying)	These operations cause lesions that accelerate the process of biological alterations	(Yahia et al., 2014)
Chemical	The richness of the date *Deglet Nour* in invertase	It causes sucrose inversion. This inversion can cause a decrease in the equilibrium of the relative humidity of dates and a change in its natural flavor	(Yahia et al., 2014)
	Sugar spotting (sugaring)	It results from crystallization of sugars in the flesh of soft date cultivars It alters fruit texture and appearance	
	Skin separation (puffiness)	The skin is dry, hard and brittle, and is separated from the flesh	
Biochemical	Enzymatic browning	The browning reaction requires the presence of O_2_, phenolic compounds and polyphenol oxidases (PPO) enzymes and is usually initiated by the enzymatic oxidation of monophenols into o-diphenols and o-diphenols into quinones, which undergo further non-enzymatic polymerization leading to the formation of pigments	(He et al., 2008)
	Nonenzymatic browning	It causes browning reactions.	(Martins et al., 2000)
Insect infestation	*Oligonychus afrasiaticus* and *O. pratensis* are mites that cause a disorder known as “BouFaroua” disorder	Affects fruit at the Hababook stage. The larvae develop around the fruit producing white filament netting, which in turn causes fruits to drop prematurely	(Yahia et al., 2014)
	*Coccotrypes dactyliperda* (date stone beetle)	the fruit dropping at the immature green stage	
	*Parlatoria blanchardii* (date palm scale)	It attacks the fruit while still green and forms white filaments around the fruit, which reduce photosynthesis and the fruits do not reach maturity.	
	*Ectomyelois ceratoniae* Zeller (date carob-moth)	It causes significant postharvest losses in stored dates. The moth is common on dates, pomegranates and carobs.	
	*Batrachedra amydraula* Meyr (lesser date moth), *Carpophilus hemipterus* (dried-fruit beetle), *C. mutilatus* (confused sap beetle), *Urophorus humeralis* (pineapple beetle), and *Haptoncus luteolus* (pineapple sap beetle),	They cause serious damage to dates on the bunch or after harvest.	
	*Vespa orientalis* (Oriental hornet), *Cadra figulilella* (raisin moth), *Aphomia sabella* Hampson (greater date moth), and the *Tyrophagus lintneri* Osborn (mushroom mite)	They infest stored dates.	
	*Ephestia cautella* Walk (fig-moth)	It is a postharvest pest that can attack dates in the orchard, packinghouses or stores. Dates at the Kimri, Khalal, and Rutab stages are not attacked by this insect, only fruits at the Tamar stage.	
	*Oryzaephilus surinamersis* L. (saw-toothed grain beetle)	It attacks stored dates	

### 2.1 Sorting:

Date fruits do not get mature at the same time, therefore, the mature fruits are generally handpicked from their bunch. The main steps of date fruit handling involve classifying, disinfection, drying, selection, grading, and packaging. The first step is the classification of the dates which aimed to remove culls and distinguish them into uniform sizes. This process can be performed by hand or by using mechanical means (Yahia, 2011). Generally, date fruits are sorted and graded manually through visual inspection by professional or guided workers (Phulpoto et al., 2012). These steps are among the most time-consuming steps in postharvest operations, which can postpone packaging and marketing of date fruits (Al Ohali, 2011). Sorting is based on the uniform sizes, maturity, flesh consistency from soft to dry, color from yellow to black, and shape. The date fruit is commonly sorted by removing culls, immature or overripe fruits, and discarding fruits with physical and physiological disorders, and abnormalities such as parthenocarpic. Color is one of the important external characteristics used in sorting and grading, it is a vital feature between acceptable or immature date fruits. Numerous studies are being conducted for the purpose to perform intelligent and precise operations with quick sorting of date fruits to improve the grading efficiency (Siddiq and Greiby, 2014). Fadel et al. (2001) envisaged a machine vision system according to sugar content and color values (RGB) to differentiate among various date cvs. Another machine vision was designed to evaluate the date quality by reflective near-infrared imaging through analyzing two-dimensional images, which improved grading accuracy (Lee et al., 2008). Pourdarbani et al. (2015) has also developed an automatic sorting system for date fruits which differentiate the Khalaal, Rutab, and Tamar according to their color and maturity.

### 2.2 Washing and grading:

The date fruits arrived from farms may be contaminated with any physical particles, i.e. dust, biological materials, i.e. plant parts, and chemical products. So, this must be eliminated before processing. Brushing or pressurized air is among the most used techniques for cleaning but it should be kept in mind that the dates are sensitive to physical damages and the cleaning should be carried softly (Yahia et al., 2014). The grading of dates is based on their size and weight. Several factors influence quality grades in date fruits such as lack of visual defects, abnormality, skin puffiness, sunburn, insect damage, uniformity of color and size, decay, fermentation, and mechanical damage. These criteria are usually applied for Codex and US Grades A, B, C, standard and substandard, for all types of date fruits including whole, pitted, or dry. The size of a package is one of the important factors for the quality criteria and depends on the date fruits variety. In the U.S. for Medjhool date fruits, there are three different gradings based on the weight i.e. for<10, 10–15, and more than 15 dates per pound categorized as Jumbo, Mixed, and Conventional respectively (Yahia and Kader, 2011). The classification may differ in different countries. For example in Pakistan, date fruits are mainly classified, from best to worst, as Extra Class, Category-A, Category-B, Good Quality, Fair Quality, and Industrial Grade (Phulpoto et al., 2012).

### 2.3 Dehydration and storage:

The optimum moisture content (23% − 25%) of the date fruits can be achieved by dehydration. This is one of the most important requirements of quality preservation during storage. The use of hot air or steam (60 to 65 °C) is a successful method for dehydration which may generally be performed in an industrial oven. The recommended duration for this process is between 4 and 8 h (Yahia et al., 2014). Studies recommended that using steam for dehydration improves resistance to microbial pathogens (Kader, 2003, Kader, 2007).

During handling and storage of date fruits, the packaging is applied to avoid water loss, physical and insect damages. There are various types and dimensions used for the packaging of date fruits. The fiberboard boxes are used for marketing 6.8 kg of date fruits. Whereas, the dry date fruits are usually exported in large reinforced cartons. A wide variety of sizes and types with different capacity are commonly used for consumer packages including transparent bags or overwrapped trays with poly film, and round fiberboard cans (0.5–1 kg), transparent plastic containers (0.2–0.3 kg), and small bags (50–60 g dates) (Yahia et al., 2014).

Before storage, the date should be cooled to < 10 °C, favorably to 0 °C. Hydro-cooling is one of the cooling methods of date fruits at the Khalal stage which requires 10–20 min at near 0 °C temperature (Elansari, 2008). Before shipping the containers of date fruits, excess moisture from the cooled date fruits should be removed from the surface. During shipping, some physiological processes like sugar crystallization can be reduced by optimum temperature and the speed of refrigeration inside the shipping containers. Also, tearing the skin of date fruits, due to the breaking of cell walls, can be facilitated in low temperatures and above 20% moisture (Glasner and Botes, 2002).

Date fruits are usually stored at low temperatures to prevent color changes, sugar spots, and syrupiness processes, disease incidence, and insect infestation. In addition, cold storage minimizes flavor, textural, and quality losses. Optimal storage temperature depends on cvs and ripening stage of date. To prevent water loss and over-ripening, date fruits at the Khalal stage should be stored at 0 °C and 85 to 95% relative humidity (RH). Tamar date fruits can be stored at 0 °C for 6–12 months. Some semi-soft cvs, like *Deglet nour* and *Halawi*, can be stored longer than soft cvs such as *Medjhool* and *Barhi*. For longer storage time, the date fruits should be kept below the highest freezing temperature (–15.7 °C). However, some dry date fruits with 20% or lower moisture can be stored at − 18 °C, 0 °C, 4 °C, and 20 °C for more than 1 year, 1 year, 8 months, and 1 month, respectively (Siddiq and Greiby, 2014). In comparison to *Deglet nour* dates stored at 0 °C, for 10 months of storage freezing at −20 °C maintain their overall quality better after a subsequent thawing of 7 days at 5 °C. Consequently, extending the shelf-life during a long-term storage period −20 °C was preferable. Both chilling and freezing could be commercially used and simply realized at an industrial scale (Jemni et al., 2019b).

Another important factor during the storage of date fruits is RH, which minimizes weight loss, the development of fungal diseases, and some physiological disorders. Appropriate RH for storage of date fruits is 65–75%, the lower RH, the greater the resistance to microbial pathogens. During storage, dates should not be kept with strong odors emitters commodities such as apples, garlic, onion, and potato because they can absorb their odors. In addition, some chemicals like sulfur dioxide (SO_2_) and NH_3_ have a detrimental impact on date quality (Kader and Awad, 2009).

### 2.4 Automation and robotics in the handling of fruits:

Numerous scientific studies had been highlighting that the availability of natural resources, mostly soil, and water, had been decreasing, where the human population, thus the need for food is increasing throughout the world. Therefore, most of those studies recommend that optimum use of natural resources with the highest outputs is necessary for ensuring sustainability on the planet. This then highlights the importance of innovation and agriculture, including advancements in technology such as sensors, robots, GPS technology, and the internet of things (IoT) (Rose and Chilvers, 2018). The increase in the need to supply products in a short time and with high quality had increased the need for the automated grading of horticultural crops. Besides that, it is well known that the damaged fruits with defects can not be stored for long time period. Thus, automatic and robotics can provide a more accurate and quicker selection of fruits than human labor. Besides that, visible-NIR (Near Infrared) spectroscopy is known to have a long history in the use for analyzing fruit color and so the quality of fresh products (De Jager and Roelofs, 1996). Besides scientific studies, the industry is at the same time working on automation in agriculture and there are several companies now available in the market which produce packing lines with multiple cameras and sensors, integrated with Vis-NIR spectrometric probes to predict different quality parameters of the fruits (Walsh et al., 2020). Moreover, automation technologies helped to introduce guarantee safety and security of food for the consumers. There are available technologies for measuring fruit size, color, shape, external defect, soluble solids content, fruit acidity, and several internal quality parameters (Njoroge et al., 2002). Automation also helps to easily record any information about the products to ensure traceability, including grower name, harvest time, agro-chemicals applied, and etc. This kind of information is nowadays being highly asked by the consumers. (Kondo, 2010) reported that automation is highly applicable in orange grading, eggplant grading, and leek preprocessing. Similar to other fruits, such kinds of techniques can be used in date processing. In a such study, Alavi (2012) reported that Mamdani fuzzy inference system can be used to provide decision-making for the classification of Mozafati date fruits. The study suggested that the evaluation based on MFIS model is more accurate (86.00%) than experts and provides better date fruits grading representation. In another study, Al Ohali (2011) suggested that a prototypical computer vision system, which works on the base of RGB images, could provide 80.00% accuracy in grading and sorting of date fruits.

### 2.5 Nanotechnology utilization in storage:

Nanotechnology is a field of research and innovation including design, characterization, production, and application of structures, devices, and systems, at the nanoscale (about 1 to 100 nm). The products of nanotechnology are reported to be used in more than hundreds of sectors including cosmetics, textile, sports, drugs, environment production, clean-up products, agriculture (including micronutrients, nano pesticides, nanomaterials-based delivery of CRISPR for crop improvement, nanosensors, nanofibers, nanocoatings, food packaging, fruit storage) and etc. (Lyddy, 2009; He et al., 2019)). Nanotechnology had been reported to be very effective as an antifungal agent in many fruits and vegetables. Coatings and films have several advantages in food preservation by reducing water loss, retardation of ripening, prevention of microbial decay, reduction of chilling and mechanical injury, and improving the visual appearance of food products including fruits and vegetables. Polysaccharides, proteins, and lipids can be used for the production of edible coatings. Chitosan-based nanoparticles are among the most widely studied and used materials for producing edible films and coatings. It is a natural antimicrobial compound known to reduce the postharvest decay of fruit and vegetables (Chaudhary et al., 2020, Kumar et al., 2020). In a most recent study, Saqib et al. (2020) recommended that the chitosan-coated iron oxide nanoparticles (CH–Fe2O3 NPs) provide successful results against *Rhizopus stolonifer* in peach fruits. Nanotechnology materials can also be used to produce innovative, active and smart packaging which can improve the storability of fruits and vegetables by controlling the respiration and transpiration of the fresh products (Acharya and Pal, 2020, Alfei et al., 2020). The use of these packaging is promising alternatives for any kind of fruit and vegetables, including date palms.

## 3 Microbial and insect effects on date palm

The microbes and insects causes severe damage to fruits particularly during heavy rains at the last stages of fruit ripening (Kader, 2009, Yahia et al., 2014). The estimated loss of the dates, caused by fungal spoilage is estimated to be more than 50% (Al-Sheikh, 2009, Atia, 2011). The most common fungi genera responsible of decay spoilage losses because of their pathogenicity are *Alternaria*, *Aspergillus*, *Cladosporium, Fusarium, Rhizopus,* and *Penicillium* (Al-Bulushi et al., 2017, Al-Mutarrafi et al., 2019, Al-Sheikh, 2009, Al Ghamdi et al., 2019, Bokhary, 2010, Colman et al., 2012, Gherbawy et al., 2012, Ibrahim and Rahma, 2009, Lobo et al., 2014, Nasser, 2017, Omamor and Hamza, 2006, Palou et al., 2016, Quaglia et al., 2020, Ragab et al., 2001).

### 3.1 Effects of mold, yeast, and bacteria on dates

There are different causes of microbial spoilage at the date fruits. The main reasons are mold, bacteria, and yeast (Al Hazzani et al., 2014, Atia, 2011, Hamad, 2008, Ibrahim and Rahma, 2009, Kader, 2007). The mold caused deterioration and fermentation leads to the development of undesirable alcoholic flavor. Due to the influance of temperature on microbial growth it can be assumed that temperature significantly influences the storability and shelf life of date fruits. Microbial growth was also reported to be facilitated by the high moisture content (Kader, 2007, Lobo et al., 2014).

*Aspergillus*, *Penicillium*, *Alternaria,* and *Fusarium* are among the most important mycotoxin-producing microbes which were previously isolated from date fruits (Hamad, 2008, Kader, 2007). *Aspergillus spp*. are the most common fungi species infecting dates under moderate temperature and high RH (Shenasi et al., 2002b).

The most important causal fungal agents of disease in Spanish date palm fruits were *P. expansum*, *A. alternata*, *C. cladosporioides*, and *A. nigerclade* (Palou et al., 2016). Other species belonging to the genera *Aspergillus*, *Penicillium,* and *Cladosporium* are *A. flavus*, *A. tubingensis*, *P. brevicompactum*, *P. crustosum*, *P. glabrum*, *P. venetum*, *C. cladosporioides*, *C. limoniforme* and *C. halotolerans* (Quaglia et al., 2020).

The spoilage of dates could be also caused by some yeast species and lactic acid development by the yeast. Previous studies recommended that the microbial counts are generally lower at the Kimri stage which significantly increases during Rutab stage and then decreases at Tamar stage (Shenasi et al., 2002a).

Storage at low temperatures reduces the incidence and severity of souring (Yahia et al., 2014). Microbial pathogens decrease when the fruits are stored at temperatures below 5 °C as compared with the 25 °C (Abdul Aly et al., 2018). The freezing at −19 °C provides better control of the microbial pathogens (Al Jasser, 2010). Overall, current knowledge suggests that refrigeration is the best way to control pathogens in stored date fruits (Abdul Aly et al., 2018). Avoiding fluctuations in temperature is among the most important methods for the prevention of microbial growth. Besides that, drying the fruits down to < 20% moisture together with sanitation are the other major methods for the control of yeast and mold. After the prohibition of methyl bromide (disinfestation agent), different alternatives had been studies in the scientific media and date palm industry. Chlorination of the washing water is one of the alternatives recommended by Beuchat et al. (2004). However further studies also recommended that chlorine can cause skin irritation and/or react with organic materials to produce carcinogens (Rico et al., 2008). Ozone application is the other well-known methods against microbial growth. Moreover, controlled atmosphere applications, physical control measures and electrochemical disinfection are also available (Dehghan-shoar et al., 2010). Bessi et al. (2014) recommended that anolyte water (with a pH 7–7.5 and oxidation–reduction potential 800–850 mV) is an ecologically safe alternative when used at 1–5% with 2 to 4 min for disinfecting dates where it was noted to provide 99.5% reduction of the total count of mesophilic bacteria, and 100% reduction of the yeast and molds. The use of edible films and/or edible coatings (gelatin, chitosan, guar gum, etc.) was noted to delay the ripening which improves the storability of the *Barhi* date fruits (Abu-Shama et al., 2020). Studies of Aloui et al. (2014) was also suggested that the combination of chitosan and locust bean gum inhibits the development of *A. flavus*. Dehydration to below 20% of moisture was also known to greatly reduce the incidence of pathogens. Current knowledge also suggest that the handling method and cold-chain significantly affects the optimum shelf life of date fruits (Kader, 2003). To sum up this topic, Al-Ahmadi et al. (2016) suggested that the best condition for the soft date fruits is cold environment, whereas the dry conditions are better for dry date fruits.

### 3.2 Insect infestation

One of the main causes of losses in date fruits is insects. *Coccotrypes dactyliperda* (date stone beetle) infect date fruits at the green stage which results in early fruit dropping at immature stage. *Parlatoria blanchardi* (date palm scale) also attacks green fruits and forms white filaments around the fruit. The infection results in a reduction of photosynthesis and the fruits do not reach maturity. The date carob-moth (*Ectomyelois ceratoniae* Zeller) is a common insect in different dates producing areas and it causes significant postharvest losses during storage, whereas, the lesser date moth (*Batrachedra amydraula* Meyr), dried-fruit beetle (*Carpophilus hemipterus*), pineapple beetle (*Urophorus humeralis*), confused sap beetle (*Carpophilus mutilates*), and pineapple sap beetle (*Haptoncus luteolus*), are also known to cause serious damage to dates on the bunch or after harvest. Some of the other insects which are harmful to date after postharvest are *Cadra figulilella* (raisin moth), *Vespa orientalis* (Oriental hornet), *Aphomia sabella* Hampson (greater date moth), and *Tyrophagus lintneri* Osborn (mushroom mite) (Bachrouch et al., 2010, Jemni et al., 2014a, Jemni et al., 2014b, Marouf et al., 2013, Mediouni et al., 2004, Mohandass et al., 2007, Wakil et al., 2015, Warner et al., 1990).

## 4 Disinfestation

Infestations in date fruits are due to insects (mainly coleopterons, lepidopterons and hymenopterons) as well as by bacteria like (*Escherichia coli, Staphylococcus aureus,* and *Bacillus cereus*) and by several fungi genera (Warner et al., 1990). Regulations on the use of agrochemicals to control pathogens became more and more stringent. In fact, the reduction in the use of postharvest insecticides needs studies on technically and economically viable sustainable substitutes for the purpose to maintain fruit quality and safety. Among the chemicals/process being used to protect dates includes the use of phosphine(PH_3_), SO_2_, carbon sulfate (CS_2_), carbon dioxide (CO_2_), ethylene oxide (C_2_H_4_O), ozone (O_3_), microwaving (MW), freezing, heat treatment, ultraviolet (UV-C) radiation, radio frequency (RF) or irradiation, alone or combined, have been stated (Jemni et al., 2015).

PH_3_ is a powerful fumigant, but it requires 3 to 5 days at 20 °C and 60% RH. The UV-C light (200–280 nm) is non-ionizing radiation with probed effects as an efficient technique, mainly at 6.22 kJ m^-2^for keeping quality of *Deglet nour* cv (Jemni et al., 2014a). Micro waves have been used to heat products by converting electromagnetic energy to heat energy and are a potential quarantine treatment method to control some insects pests (Dorantes-Alvarez et al., 2000, Ooi et al., 2002, Zouba et al., 2009) RF energy quickly heat insect larvae, which can kill the insects with a negligible influence on fruit quality (Monzon et al., 2006). The insecticidal effect of O_3_ is due to its ability to diffuse through biological cell membranes and high oxidation potential (Jemni et al., 2015). Electrolyzed water (EW) is a more effective bactericide and fungicide than NaClO while it enters more easily in the product surface (AL-Haq et al., 2005, Izumi, 1999, Martínez-Hernández et al., 2013, Tomás-Callejas et al., 2011). Neutral EW (pH 7.2, ORP 814 mV, and 300 mg L^−1^of free NaClO) was very effective against *Ectomyelois ceratoniae* spread on *Deglet nour* date, being considered as a hopeful tool for commercial disinfection and extending shelf-life (Jemni et al., 2014b).The combined use of UV-C light and EW or ozonated water lowered the microbial growth on *Deglet nour* dates which showed a better color and sensorial quality than control (Jemni et al., 2014a). The ionizing radiation is also an effective disinfestations method that has negligible effects on fruit quality (Çopur and Tamer, 2014, Yahia et al., 2014)

The modified atmosphere packaging (MAP) technique alters the gases surrounding perishable food products creating a modified/adequate atmospheric composition for the products. The MAP maintains a high RH around the produce and slow senescence. High CO_2_levels in MAP inhibits the growth of many fresh food spoilage microorganism (Al-Ati and Hotchkiss, 2002, Artés, 1995, Artés et al., 2006, Escalona et al., 2004, Farber et al., 2003, Homayouni et al., 2015, Jemni et al., 2016a, Sandhya, 2010, Toivonen et al., 2009). The combined use of CO_2_ and PH_3_ provides better performance with shortened duration of control of the pests (*Oryzaephilus surinamensis* and *Tribolium confusum*) in stored dates without causing undesirable quality changes (El-Mohandes, 2010). The O_2_ promotes several deteriorative reactions in foods including fat and pigment oxidation, browning reactions, and is highly required for pathogens (Al-Ati and Hotchkiss, 2002, Farber et al., 2003, Sandhya, 2010).

## 5 The overview of physiological processes in date palm which affects its storage potential

### 5.1 Respiratory activity

Date fruits have a relatively low respiration rate (RR) and are classified as non-climacteric fruits. However, some of the previous studies with Zahdi, Derey, Sultan and Braim cvs claimed the date fruits as climacteric (Kader and Yahia, 2011, Lobo et al., 2014). Respiratory activity varies depending on several factors: temperature, ripening stage, moisture content, CO_2_, O_2_, and C_2_H_4_ levels within the package. Commonly, the RR is high initially but decreases progressively until reaching the lowest position. At that time the fruits called in physiologically mature and then the RR increases to a slight peak as the fruit ripens (Kader and Yahia, 2011). At 20 °C the RR is lower than25 mLCO_2_kg^−1^h^−1^ for Khalal stage dates, and then 5 mL CO_2_ kg^−1^h^−1^ for Rutab and Tamar stages (Lobo et al., 2014). Very few and incomplete works are available on the RR and C_2_H_4_ emission from date fruits. However, both are key factors in the design and operation of refrigerated storage facilities, since they affect chilling storage and transportation, air exchange and circulation needs, loading density, and handling, packaging, and stacking methods. Particularly, the RR values are needed for an optimum design of polymeric packages when MAP takes place (Artés et al., 2002, Jemni et al., 2016b).

**Fig. 4:**
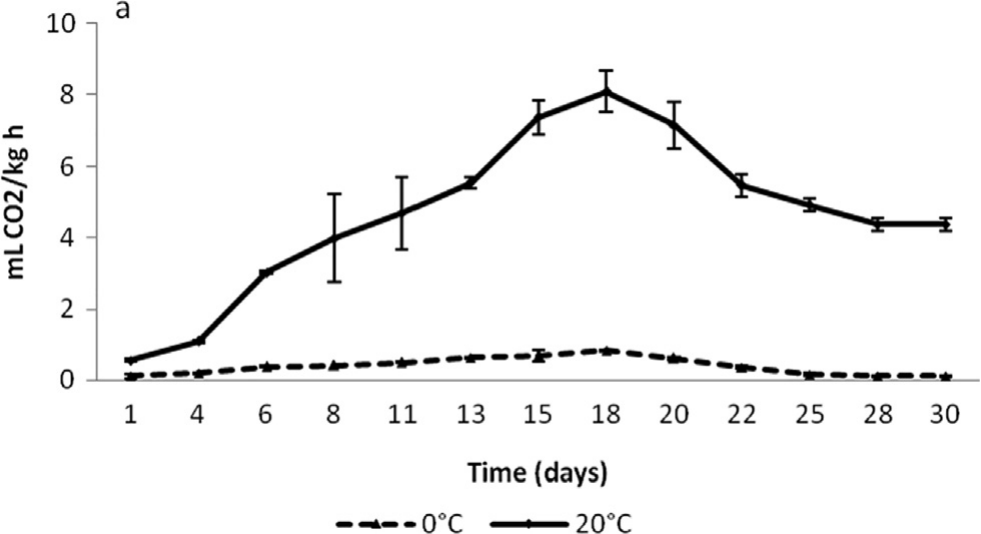
Changes in respiration rate of *Deglet nour* cv at Tamar stage with 20.13 ± 1.35% moisture content at 0 and 20 °C (Jemni et al., 2019b).

Changes in RR of *Deglet nour cv* (moisture 20.13 ± 1.35%) at 0 °C and 20 °C throughout storage are shown in Fig. 4. At 0 °C, the RR was 0.10 ± 0.03 mL CO_2_ kg^−1^h^−1^, with a maximum value of 0.83 ± 0.02 mL CO_2_kg^−1^h^−1^. At 20 °C, the RR increased from 0.55 ± 0.04 mL CO_2_kg^−1^h^−1^ in the initial day up to 8.1 ± 0.58 mL CO_2_ kg^−1^h^−1^ in day 8. This increase was very probably due to fungal attacks which were first observed after 6 days of storage. The presence of mold, yeast, and bacteria on the dates increases the RR and the need for aeration during storage. Cured *Deglet nour* dates with 20 to 22% moisture kept at 24 °C produce about 0.22 mLCO_2_kg^−1^h^−1^) and about 1.1 mL CO_2_/kg^−1^h^−1^) when moisture was 27% (Jemni et al., 2019b, Rygg, 1975).

### 5.2 Ethylene production

**Fig. 5:**
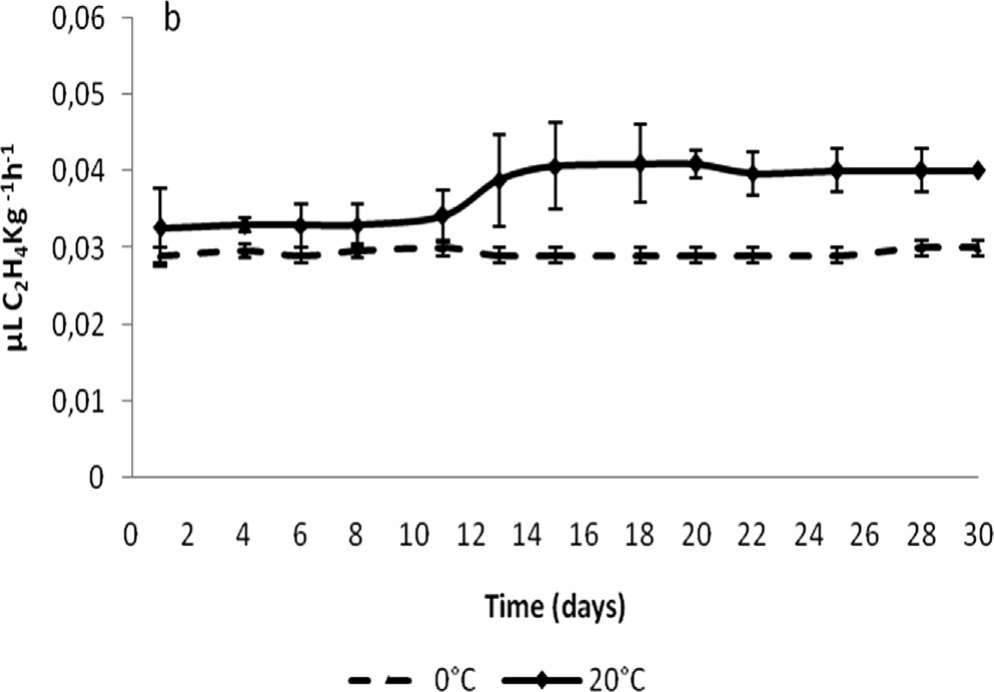
Changes in ethylene emission of *Deglet nour* cv at Tamar stage with 20.13 ± 1.35% moisture content at 0 and 20 °C (Jemni et al., 2019b).

Ethylene (C_2_H_4_) is an important plant hormone that significantly influences the changes in fruit firmness, color, soluble solids concentration, and titratable acidity in dates. The production rate of C_2_H_4_ at dates changes according to the stages. It is reported to be < 0.5 µL kg^−1^h^−1^ for Khalal stage (at 20 °C), and < 0.1 µL kg^−1^h^−1^ for Rutab and Tamar stages. Studies with Halawi variety suggested that the C_2_H_4_ production was zero for 91 days after pollination, which then starts to increase (reaching a peak in 15 days) and then having a declining trend. Rutab and Tamar stage of dates is reported to be not influenced by exposure to C_2_H_4_ (Kader and Yahia, 2011). The C_2_H_4_ emission of *Deglet nour* cv within packages stored at 0 °C was quite stable at 0.029 ± 0.003 µL kg^−1^h^−1^ (Fig. 5). Also, no relevant changes in C_2_H_4_ emission within packages at 20 °C were found which values ranging between 0.033 ± 0.005 and 0.041 ± 0.006 µL kg^−1^h^−1^. The slight or no significant rise in C_2_H_4_ started about at the same time as that in CO_2_ production, the authors indicated that it was probably due to fungal development (Jemni et al., 2019b). The changes in RR and C_2_H_4_ emission were typical of a non-climacteric fruit and the reached RR levels might be considered as moderate (Kader and Kasmire, 1984).

## 6 Nutrients composition in date palm

Date fruit is very nutritious and has assimilative components (Biglari et al., 2009). Its 2762  kcal kg^−1^ average energy is higher than many other food sources, i.e. figs 2553, wheat bread 2464, catfish 2294, avocado 1786, milk (3.3% fat) 1345, cooked rice 1198, banana 924, mango 652, apple 587, apricot 486 or orange 473 kcal kg^−1^ (Gebhardt and Thomas, 2002). About 65–70% of the date fruit is composed of carbohydrates mostly formed by inverted forms, glucose, and fructose (Ghnimi et al., 2017). The level of sucrose is highly variable and mainly depended upon the cvs. with very low (5.1%) for *Mabrrom* (Ghnimi et al., 2017) or very high (56.6%) for *Deglet nour* cv (Kchaou et al., 2013). The carbohydrate levels of *Burni*, *Suqaey,* and *Khodari* cvs were 81.4%, 79.7%, and 79.4% respectively (Assirey, 2015). In the same way, the carbohydrate content of 9 different cvs was reported to be between 59.6% and 76.8% (Abdul-Hamid et al., 2020). These results highlight the varietal dependences on the carbohydrate level. The levels of sucrose, glucose, and fructose are reduced after a heat treatment prior to storage (Ben-Amor et al., 2016a).

The protein composition of different date palm cvs was reported by Assirey (2015) to vary between 1.72% (*Mabroom*) to 4.73% (*Shlaby*). Similar ranges of proteins in different date cvs were found by Al-Harrasi et al., 2014, Abdul-Hamid et al., 2020as 2.63–3.78% and 2.08–3.1%, respectively. The amino acid and protein composition of the dates vary among cvs and ripening stages. Assirey (2015) reported 18 amino acids where the highest levels were for glutamic acid (158–265 mg 100 g^-1^dw), aspartic acid (127–225 mg 100 g^-1^dw), proline (86–113 mg 100 g^-1^dw), glycine (83–102 mg 100 g^-1^dw) and alanine (78–105 mg 100 g^-1^dw). The other amino acids were arginine, cysteine, histidine, isoleucine, leucine, lysine, methionine, phenylalanine, serine, threonine, tryptophan, tyrosine, and valine. Among these 18 amino acids, 17 were also reported by Hamad et al. (2015)except threonine; and 17 of them were also found by Ali et al. (2014) except tryptophan. The amino acid contents may exhibit different reactions against the postharvest treatments and storage. For example, the glutamic acid and methionine were unaffected by storage duration but reported to be affected by hot water treatment (Ben-Amor et al., 2016b).

Dietary fiber is a carbohydrate that cannot be completely broken down by human digestive enzymes and is typically comprised of polysaccharides such as cellulose, beta-glucan, resistant starch, pectin, and arabinoxylans (Hussain et al., 2020). Numerous studies have reported high contents of dietary fiber for date fruits (Camire and Dougherty, 2003, Englyst and Hudson, 1996, Prosky et al., 1988). Mrabet et al. (2017) found that the dietary fiber level of the Tunisian date palm cvs ranged from 4.7 to 7 g 100 g^−1^, and the oil- and water-retention capacities were 4 and 17 mL g^−1^ fiber, respectively. This work showed that the dietary fiber composition of neutral Klason consists of 33.3–50.4% lignin, 17.0–24.8% cellulose, 10.7–16.7% uronic acid, and 15.6–25.7% monosaccharides. The composition and concentration of dietary fiber vary depending on the cvs and also on the ripening stage of the date fruits. For example (Mustafa et al., 1986) reported that the Rutab dates had higher pectin content than Tamar fruits, due to the minimal activity of pectin esterase during this period. Date fruits with a high amount of pectin and a low amount of lignin are accepted as high-quality, where the opposite pattern is noted as inedible. The *Deglet nour* and *Allig* cvs were reported to have higher dietary fiber levels than those of Tunisian cvs (14.4% and 18.4%, respectively) (Elleuch et al., 2008).

Phenolic compounds are secondary metabolites that are responsible for the red color of the date fruits and have strong antioxidant properties. The total phenolic compounds content (TPC) of date fruit vary depending on the cv, ripening state, and growing conditions (Daoud et al., 2019, El Hadrami et al., 2011, Saafi et al., 2009). Date fruits are known to have high antioxidant, anti-inflammatory, and anti-cancer properties and to stimulate the immune system by the valuable amounts of nutraceuticals contained (Hussain et al., 2020, Khalid et al., 2017). El Hadrami et al. (2011) reported that the polyphenols level decreased as the fruits ripen and can only be increased if the fruits are damaged. Abu-Reidah et al. (2017)tested 52 different phenolic compounds on pulp, skin, frond, cluster, and pollens of date palms showing that only 3 of them (L-7-(6Rh)Hx, I-3-(6Rh)Hx, and Chr-7-(6Rh)Hx) are found on all plant parts and 17 of them are found on fruit pulp. The most abundant phenolic compounds in date palm fruit are caffeic, gallic, cinnamic, and ferulic acids (Bouhlali et al., 2018, Daoud et al., 2019, Eid et al., 2013, El Hadrami et al., 2011, Hamad et al., 2015, Mrabet et al., 2016, Saafi et al., 2009). Date fruits have some soluble tannins but their levels are found to decrease as the fruits ripen (Hammouda et al., 2013). The health benefits of soluble tannins are lower than those of the other phenolic compounds (Pompella et al., 2014). The TPC of different date cvs was between 2.49 and 8.36 mg GAE g^−1^(Abdul-Hamid et al., 2020, Mansouri et al., 2005). It was also found that the sugar content of dates has a positive correlation with the TPC of fruits (Abdul-Hamid et al., 2020, Al-Farsi et al., 2005). The phenolics composition of 10 Algerian date cvs was tested on a fresh weight basis (Benmeddour et al., 2013) showing that the TPC contents varied from 167 in *Deglet nour* to 709 mg GAE 100 g^−1^ of fresh weight in Ghazi. Saafi et al. (2009) found a range from 209 to 448 mg GAE 100 g^−1^.

Date fruit is a good source of K, Ca, Mg, β-carotene, and vitamin A, with a very low content of Na and fat (USDA, 2020). Phytochemical and nutritional composition of the date fruits vary depending on the cvs, environmental condition, and ripening stage (Hussain et al., 2020, Kahramanoglu and Usanmaz, 2019, USDA, 2020). The moisture content losses during storage improve the storability of the date fruits but reduce their acceptability by the consumers (Assirey, 2015). It was reported to change from 15 to 30% in date (Gebhardt and Thomas, 2002).

The seed of the date palm have also high nutritive characteristics. The chemical composition of the date seeds includes about 1.77–9.55% total sugar, 3.7–6.14% protein, 1.07–1.30% ash, and 4.68–7.96% lipid (Bouhlali et al., 2017, Juhaimi et al., 2012). The difference in the chemical composition is due to differences among cvs, origin, ripening stage, and growing and climatic conditions (Golshan Tafti et al., 2017). The oil of date seeds is used in medicine, cosmetics and food products (Jemni et al., 2019a) and was reported to have 1.06–2.10 mg KOH g^−1^ acid value and a range of 10.1 to 25 meq O_2_ kg^−1^ peroxide value. It has been found that among 16 different fatty acids from the date seeds oleic is one of the most abundant and known to have positive impacts on human health by reducing the LDL cholesterol and cardiovascular disease risks (Jemni et al., 2019a). The negligible amounts of linoleic acid content in date seeds make it relatively stable to oxidative deterioration and good for human skin (Nehdi et al., 2018).

The storage conditions influence the chemical composition of the date including the textural quality. In fact, high temperature, especially during the retail period, reduces fruit firmness. The optimal storage temperature is highly variable among cvs (Ben-Amor et al., 2016a). A general recommendation for Tamar dates is 0 °C which helps to keep quality about 6–12 months (Kader and Awad, 2009). The combined passive MAP (6 kPa O_2_ + 12 kPa CO_2_) and moderate temperature (20 °C) may help to protect commercial quality; but the better conditions for 1 month of storage is 0 °C with or without passive MAP (Jemni et al., 2016b). It seems to exists a positive correlation between date quality and CO_2_levels around the fruits, and the use of MAP and partial vacuum improves its postharvest storability (Jemni et al., 2016a). A hot air treatment (55 °C for 30 min or 60 °C for 15 min) was effective in both controlling postharvest infestation by *Ectomyelois ceratoniae* and improve storability of *Deglet nour* cv at 2 °C (Ben-Amor et al., 2016a).

## 7 Disorders

The date fruit has some pre- and post-harvest physiological disorders which affect their quality. The most important are the white nose, black nose, and freezing damage (Chao and Krueger, 2007, Kader and Yahia, 2011). White-nose mainly appears after dry weather during the early Rutab period and causes rapid maturation of the fruits. The postharvest effect of this disorder appears as a whitish drying at the calyx end of the fruit and an increase in sugar content. The black nose is the abnormally shriveling and darkening of the fruit tip, mainly caused by the humid weather at the Khalal stage. Fruit bagging with brown wrapping paper was reported to inhibit black nose disorder (Zaid et al., 1999). The freezing damage occurs by moisture freezing inside the cells with crystallization of water (Kader and Yahia, 2011). Another important disorder of date palm fruits is wilting disorder. It is mainly caused by the high temperature and low relative humidity, which is more prevalent in recent years because of climate change. A study of Alikhani-Koupaei et al. (2018) suggested that regulated deficit irrigation can be used to control wilting disorder and fruit dropping percent.

### 7.1 Chilling and freezing injuries

Chilling and freezing injuries affect many fruits and vegetables. Chilling injury (CI) is generally a problem for tropical and subtropical fruits which are very sensitive to CI. CI mainly occurs at temperatures below 12 °C but above freezing point (0 °C). With the low, but nonfreezing temperatures, the tissues weakened leading to cellular dysfunctions because they are unable to carry on normal metabolic processes. The symptoms of CI include surface pitting, loss of flavor, internal discoloration, water soaking of the tissue, and increased susceptibility to microbial decay (Wang, 1989) Symptoms of CI may occur in 1 week to 3 months, depending on the produce, but mainly appear after the fruits are transferred to non-chilling temperatures. The use of intermitted warming, edible films/coatings, packaging, and application of salicylic & jasmonic acids are among the most used techniques for the prevention of CI (Aghdam and Bodbodak, 2013). It has been reported that date fruits are resistant to chilling injuries and low sensitivity to freezing injuries (Al-Ani, 1985, Aleid, 2012). The other type of injury is freezing which may occur in the field or in the cold rooms at a temperature below 0 °C. Exposure to freezing temperatures may cause ice formation in the tissues which results in tissue damages. Regarding freezing damages, it occurs by moisture freezing inside the cells and it could harm the dates when stored below −18 °C (Zaid et al., 1999).

### 7.2 Antioxidant activity

Lipid peroxidation is the oxidative degradation of the lipids where free radicals steal electrons from the neighboring lipids in the cell membranes (Ahmad et al., 2010, Ahmad et al., 2008, Kohli et al., 2019). This oxidation process produces more free radicals, thereby leading to chain reactions that may damage the cells (Ahmad et al., 2010, Ahmad et al., 2008, Ahmad et al., 2019, Kohli et al., 2019). Antioxidants are bio-compounds able to inhibit oxidation and terminate these chain reactions scavenging free radicals (Ahmad et al., 2010, Ahmad et al., 2008, Kohli et al., 2019). Date fruits are rich in polyphenols, carotenoids, tannins, and vitamin A which have high antioxidant potential (Alhaider et al., 2017, Benmeddour et al., 2013, Julia et al., 2015, Khalid et al., 2017, Lobo et al., 2014, Martín-Sánchez et al., 2014, Moss and Ramji, 2016). The scavenging of free radicals prevents the occurrence and/or cure some chronic diseases, i.e.: cancer, cardiovascular disease, and Alzheimer’s disease (Kim et al., 2015). The ripening stage influences the antioxidant activity of the date palm, where Khalal stage is known to have the highest antioxidant potential which reduces as fruits ripen (Mohamed Lemine et al., 2014).

## 8 Marketing

Unlike other subtropical or tropical fruits, the date fruits are not marketed as value-added products. Generally, dates are sold as a whole, ground, chopped, and pitted pieces in pastries (Al-Abid et al., 2006) or derived products like alcoholic beverages, vinegar (Huntrods, 2011), liquid sugar, jelly (Masmoudi et al., 2010) and Tamar juice (Aleid et al., 2013, Augstburger et al., 2002, Hui, 2008). Date syrup is usually obtained from very soft or low-quality dates after hydration and concentration to 30/35°Brix which can be a good replacement of sugar in food formulations as it is a nice source of fructose and glucose (Tavakolipour and Kalbasi-ashtari, 2007). The date fruits collected at the ripening stage can be handled differently. Kimri stage date with green color is marketed for pickles and chutney, Rutab stage can be used for butter, jam, and paste, Khalal stage is used for syrup and jam, and Tamar stage date is usually used into syrup, paste, and bars. By-products and other processed dates with low quality are widely used to produce ethanol, citric acid, alcohols, vinegar, and yeast in the bakery (Aleid, 2011). The dates at ripening stages are usually immersed in 3 to 4% of acetic acid or vinegar for astringency removal. Additionally, immature dates can be dipped in hot water or incubated at 32–38 °C for a few days to become soft, translucent, and better flavor (Yahia et al., 2014). Some by-products such as date seed and press cake can be obtained. Due to their high fiber contents and nutritional values, date seed can be used for feeding cattle, poultry, sheep, and camel (Amany et al., 2012). Moreover, the hearts and terminal buds of date palm trees are good additional recipe for a tasty salad.

## 9 Conclusion

Date fruits are subject to important commercial activity. However, its sensitivity to the alteration and the lack of mastery of conservation methods on production sites may cause serious problems to cultivators and operators. In this review, we have identified and discussed different methods to be used for the protection and storage of dates fruits.

The need for the preservation of date fruits is encouraged by a number of factors that make proper application of appropriate processing techniques, packaging, and storage. The suitable storage and packaging can help in: i) the preservation of dates for long term storage and uptime during periods of high consumption; ii) ensuring a higher added value and therefore increase the economic profitability for date farmers; iii) minimizing post-harvest losses of dates caused by different alterations incurred in farmers' incomes and living standards; iv) consumption of dates through good quality fruit and available throughout the year. Thus this review can be used by cultivators and operators to get updated information in the area.

## Declaration of Competing Interest

The authors declare that they have no known competing financial interests or personal relationships that could have appeared to influence the work reported in this paper.

## References

[b0005] Abdul-Hamid N.A., Mustaffer N.H., Maulidiani M., Mediani A., Ismail I.S., Tham C.L., Shadid K., Abas F. (2020). Quality evaluation of the physical properties, phytochemicals, biological activities and proximate analysis of nine Saudi date palm fruit varieties. Journal of the Saudi Society of Agricultural Sciences.

[b0010] Abdul Aly, A., Al Abid, A., Alshwakir, A., Hassan, R., Al Fahaid, Y., Ben-Salah, M., 2018. Study of the Effect of Storage Temperature on Microbial Stored Dates under Vacuum, The Sixth International Date Palm Conference, Abu Dhabi, United Arab Emirates.

[b0015] Aboryia M., Omar A.S. (2020). Effectiveness of Some Edible Coatings on Storage Ability of Zaghloul Date Palm Fruits. Journal of Plant Production.

[b0020] Abu-Reidah I.M., Gil-Izquierdo Á., Medina S., Ferreres F. (2017). Phenolic composition profiling of different edible parts and by-products of date palm (Phoenix dactylifera L.) by using HPLC-DAD-ESI/MSn. Food Research International.

[b0025] Abu-Shama H.S., Abou-Zaid F.O.F., El-Sayed E.Z. (2020). Effect of using edible coatings on fruit quality of Barhi date cultivar. Scientia Horticulturae.

[b0030] Acharya A., Pal P.K. (2020). Agriculture nanotechnology: Translating research outcome to field applications by influencing environmental sustainability. NanoImpact.

[b0035] Aghdam M.S., Bodbodak S. (2013). Physiological and biochemical mechanisms regulating chilling tolerance in fruits and vegetables under postharvest salicylates and jasmonates treatments. Scientia Horticulturae.

[b0040] Ahmad P., Jaleel C.A., Salem M.A., Nabi G., Sharma S. (2010). Roles of enzymatic and nonenzymatic antioxidants in plants during abiotic stress. Critical reviews in biotechnology.

[b0045] Ahmad P., Sarwat M., Sharma S. (2008). Reactive oxygen species, antioxidants and signaling in plants. Journal of Plant Biology.

[b0050] Ahmad P., Tripathi D.K., Deshmukh R., Pratap Singh V., Corpas F.J. (2019). Revisiting the role of ROS and RNS in plants under changing environment. Environmental and Experimental Botany.

[b0055] Al-Abid M., Al-Shoaily K., Al-Amry M., Al-Rawahy F. (2006). Maintaining the soft consistency of date paste. III International Date Palm Conference.

[b0060] Al-Ahmadi S.S., Ibrahim R.A., Ouf S.A. (2016). Application of ozone to control insect pests and moulds of date fruits. Biosciences Biotechnology Research Asia.

[b0065] Al-Ani A. (1985). Post harvest physiology of horticultural crops.

[b0070] Al-Ati T., Hotchkiss J.H. (2002). Application of packaging and modified atmosphere to fresh-cut fruits and vegetables. Fresh-cut fruits and vegetables: science, technology, and market.

[b0075] Al-Bulushi I.M., Bani-Uraba M.S., Guizani N.S., Al-Khusaibi M.K., Al-Sadi A.M. (2017). Illumina MiSeq sequencing analysis of fungal diversity in stored dates. BMC microbiology.

[b0080] Al-Farsi M., Al-Amri M., Al-Rawahi F., Al-Abid M., Gohs U. (2010). Disinfestation of dates using electron beams in comparison with other treatments. IV International Date Palm Conference.

[b0085] Al-Farsi M., Alasalvar C., Morris A., Baron M., Shahidi F. (2005). Comparison of antioxidant activity, anthocyanins, carotenoids, and phenolics of three native fresh and sun-dried date (Phoenix dactylifera L.) varieties grown in Oman. Journal of agricultural and food chemistry.

[b0090] AL-Haq, M.I., Sugiyama, J., Isobe, S., 2005. Applications of electrolyzed water in agriculture & food industries. Food Science and Technology Research 11, 135-150.

[b0095] Al-Harrasi A., Rehman N.U., Hussain J., Khan A.L., Al-Rawahi A., Gilani S.A., Al-Broumi M., Ali L. (2014). Nutritional assessment and antioxidant analysis of 22 date palm (Phoenix dactylifera) varieties growing in Sultanate of Oman. Asian Pacific journal of tropical medicine.

[b0100] Al-Mssallem I.S., Hu S., Zhang X., Lin Q., Liu W., Tan J., Yu X., Liu J., Pan L., Zhang T. (2013). Genome sequence of the date palm Phoenix dactylifera L. Nature communications.

[b0105] Al-Mutarrafi M., Elsharawy N.T., Al-Ayafi A., Almatrafi A., Abdelkader H. (2019). Molecular identification of some fungi associated with soft dates (Phoenix dactylifera L.) in Saudi Arabia. Advancement in Medicinal Plant Research.

[b0110] Al-Redhaiman K. (2005). Chemical changes during storage of'Barhi'dates under controlled atmosphere conditions. HortScience.

[b0115] Al-Sheikh H. (2009). Date-palm fruit spoilage and seed-borne fungi of Saudi Arabia. Res J Microbiol.

[b0120] Al Ghamdi F.L., Bokhari F.M., Aly M.M. (2019). Toxigenic fungi associated with dried Fruits and fruit-based products collected from Jeddah province. Journal Of Pharmacy And Biological Sciences.

[b0125] Al Hazzani A.A., Shehata A.I., Rizwana H., Moubayed N.M., Alshatwi A.A., Munshi A., Elgaaly G. (2014). Postharvest fruit spoilage bacteria and fungi associated with date palm (Phoenix dactylifera L) from Saudi Arabia. African Journal of Microbiology Research.

[b0130] Al Jasser M.S. (2010). Effect of storage temperatures on microbial load of some dates palm fruit sold in Saudi Arabia market. African Journal of Food Science.

[b0135] Al Ohali Y. (2011). Computer vision based date fruit grading system: Design and implementation. Journal of King Saud University-Computer and Information Sciences.

[b0140] Alavi N. (2012). Date grading using rule-based fuzzy inference system. Journal of Agricultural Technology.

[b0145] Aleid S.M. (2011). Industrial biotechnology: date palm fruit applications. Date palm biotechnology. Springer.

[b0150] Aleid S.M. (2012). Tropical and Subtropical Fruits: Postharvest Physiology, Processing and Packaging.

[b0155] Aleid S.M., Dolan K., Siddiq M., Jeong S., Marks B. (2013). Effect of low-energy X-ray irradiation on physical, chemical, textural and sensory properties of Dates. International journal of food science & technology.

[b0160] Alfei S., Marengo B., Zuccari G. (2020). Nanotechnology application in food packaging: A plethora of opportunities versus pending risks assessment and public concerns. Food Research International.

[b0165] Alhaider I.A., Mohamed M.E., Ahmed K., Kumar A.H. (2017). Date palm (Phoenix dactylifera) fruits as a potential cardioprotective agent: The role of circulating progenitor cells. Frontiers in pharmacology.

[b0170] Ali H.S.M., Alhaj O.A., Al-Khalifa A.S., Brückner H. (2014). Determination and stereochemistry of proteinogenic and non-proteinogenic amino acids in Saudi Arabian date fruits. Amino acids.

[b0175] Alikhani-Koupaei M., Fatahi R., Zamani Z., Salimi S. (2018). Effects of deficit irrigation on some physiological traits, production and fruit quality of ‘Mazafati’date palm and the fruit wilting and dropping disorder. Agricultural Water Management.

[b0180] Aloui H., Khwaldia K., Licciardello F., Mazzaglia A., Muratore G., Hamdi M., Restuccia C. (2014). Efficacy of the combined application of chitosan and Locust Bean Gum with different citrus essential oils to control postharvest spoilage caused by Aspergillus flavus in dates. International journal of food microbiology.

[b0185] Amany M., Shaker M.A., Abeer A. (2012). Antioxidant activities of date pits in a model meat system. International Food Research Journal.

[b0190] Artés F. (1995). Innovaciones en los tratamientos físicos modulados para preservar la calidad de los productos hortofrutícolas en la postrecolección. III. Tratamientos gaseosos. Revista española de ciencia y tecnología de alimentos.

[b0195] Artés F., Escalona V., Artés-Hdez F. (2002). Quality and physiological changes of fennel under controlled atmosphere storage. European Food Research and Technology.

[b0200] Artés, F., Gómez, P.A., Artés-Hernández, F., 2006. Modified atmosphere packaging of fruits and vegetables.

[b0205] Ashraf Z., Hamidi-Esfahani Z. (2011). Date and date processing: a review. Food reviews international.

[b0210] Assirey E.A.R. (2015). Nutritional composition of fruit of 10 date palm (Phoenix dactylifera L.) cultivars grown in Saudi Arabia. Journal of Taibah University for science.

[b0215] Atia M. (2011). Efficiency of physical treatments and essential oils in controlling fungi associated with some stored date palm fruits. Aust J Basic Appl Sci.

[b0220] Augstburger F., Berger J., Censkowsky U., Heid P., Milz J., Streit C. (2002). Organic Farming in the Tropics and Subtropics: Date Palm.

[b0225] Awad M.A. (2007). Increasing the rate of ripening of date palm fruit (Phoenix dactylifera L.) cv. Helali by preharvest and postharvest treatments. Postharvest Biology and Technology.

[b0230] Bachrouch O., Jemâa J.M.-B., Wissem A.W., Talou T., Marzouk B., Abderraba M. (2010). Composition and insecticidal activity of essential oil from Pistacia lentiscus L. against Ectomyelois ceratoniae Zeller and Ephestia kuehniella Zeller (Lepidoptera: Pyralidae). Journal of stored products research.

[b0235] Barreveld, W., 1993. Date palm product. Rome, Italy. FAO Agricultural Services Bulletin 101, 211p.

[b0240] Ben-Amor R., de Miguel-Gómez M.D., Martínez-Sánchez A., Aguayo E. (2016). Effect of hot air on Deglet Noor palm quality parameters and on Ectomyelois ceratoniae. Journal of Stored Products Research.

[b0245] Ben-Amor R., Dhouibi M.H., Aguayo E. (2016). Hot water treatments combined with cold storage as a tool for Ectomyelois ceratoniae mortality and maintenance of Deglet Noor palm date quality. Postharvest Biology and Technology.

[b0250] Benmeddour Z., Mehinagic E., Le Meurlay D., Louaileche H. (2013). Phenolic composition and antioxidant capacities of ten Algerian date (Phoenix dactylifera L.) cultivars: a comparative study. Journal of Functional Foods.

[b0255] Bessi H., Debbabi H., Grissa K., Bellagha S. (2014). Microbial reduction and quality of stored date fruits treated by electrolyzed water. Journal of food quality.

[b0260] Beuchat L.R., Adler B.B., Lang M.M. (2004). Efficacy of chlorine and a peroxyacetic acid sanitizer in killing Listeria monocytogenes on iceberg and romaine lettuce using simulated commercial processing conditions. Journal of food protection.

[b0265] Biglari F., AlKarkhi A.F., Easa A.M. (2008). Antioxidant activity and phenolic content of various date palm (Phoenix dactylifera) fruits from Iran. Food chemistry.

[b0270] Biglari F., AlKarkhi A.F., Easa A.M. (2009). Cluster analysis of antioxidant compounds in dates (Phoenix dactylifera): Effect of long-term cold storage. Food chemistry.

[b0275] Bokhary H. (2010). Seed-borne fungi of date-palm, Phoenix dactylifera L. from Saudi Arabia. Saudi Journal of Biological Sciences.

[b0280] Botes A., Zaid A. (1999). Chapter III The economic importance of date production and international trade. FAO plant production and protection papers.

[b0285] Bouhlali, E.d.T., Alem, C., Ennassir, J., Benlyas, M., Mbark, A.N., Zegzouti, Y.F., 2017. Phytochemical compositions and antioxidant capacity of three date (Phoenix dactylifera L.) seeds varieties grown in the South East Morocco. Journal of the Saudi Society of Agricultural Sciences 16, 350-357.

[b0290] Bouhlali, E.d.T., El Hilaly, J., Ennassir, J., Benlyas, M., Alem, C., Amarouch, M.-Y., Filali-Zegzouti, Y., 2018. Anti-inflammatory properties and phenolic profile of six Moroccan date fruit (Phoenix dactylifera L.) varieties. Journal of King Saud University-Science 30, 519-526.

[b0295] Camire M.E., Dougherty M.P. (2003). Raisin dietary fiber composition and in vitro bile acid binding. Journal of Agricultural and Food Chemistry.

[b0300] Chao C.T., Krueger R.R. (2007). The date palm (Phoenix dactylifera L.): overview of biology, uses, and cultivation. HortScience.

[b0305] Chaudhary S., Kumar S., Kumar V., Sharma R. (2020). Chitosan nanoemulsions as advanced edible coatings for fruits and vegetables: Composition, fabrication and developments in last decade. International journal of biological macromolecules.

[b0310] Colman S., Spencer T., Ghamba P., Colman E. (2012). Isolation and identification of fungal species from dried date palm (Phoenix dactylifera) fruits sold in Maiduguri metropolis. African Journal of Biotechnology.

[b0315] Çopur Ö.U., Tamer C.E., Malik A., Erginkaya Z., Ahmad S., Erten H. (2014). Fruit Processing. Food Processing: Strategies for Quality Assessment.

[b0320] Daoud A., Malika D., Bakari S., Hfaiedh N., Mnafgui K., Kadri A., Gharsallah N. (2019). Assessment of polyphenol composition, antioxidant and antimicrobial properties of various extracts of Date Palm Pollen (DPP) from two Tunisian cultivars. Arabian Journal of Chemistry.

[b0325] Dawson W. (1982). Production and Preservation of date.

[b0330] De Jager A., Roelofs F. (1996). Prediction of optimum harvest date of Jonagold. Determination and prediction of optimum harvest date of apples and pears. COST.

[b0335] Dehghan-shoar Z., Hamidi-esfahani Z., Abbasi S. (2010). Effect of temperature and modified atmosphere on quality preservation of Sayer date fruits (Phoenix dactylifera L.). Journal of food processing and preservation.

[b0340] Dorantes-Alvarez L., Barbosa-Cánovas G.V., Gutiérrez-López G., Barbosa-Cánovas G.V., Gould G.W. (2000). Blanching of fruits and vegetables using microwaves. Innovations in food processing.

[b0345] Eid N.M., Al-Awadi B., Vauzour D., Oruna-Concha M.J., Spencer J.P. (2013). Effect of cultivar type and ripening on the polyphenol content of date palm fruit. Journal of agricultural and food chemistry.

[b0350] El-Mohandes M. (2010). Methyl bromide alternatives for dates disinfestations. IV International Date Palm Conference.

[b0355] El Hadrami A., Daayf F., Elshibli S., Jain S.M., El Hadrami I., Jain S.M., Al-khayri J.M., Johnson D.V. (2011). Somaclonal variation in date palm. Date palm biotechnology.

[b0360] Elansari A. (2008). Hydrocooling rates of Barhee dates at the Khalal stage. Postharvest biology and technology.

[b0365] Elleuch M., Besbes S., Roiseux O., Blecker C., Deroanne C., Drira N.-E., Attia H. (2008). Date flesh: Chemical composition and characteristics of the dietary fibre. Food chemistry.

[b0370] Englyst H.N., Hudson G.J. (1996). The classification and measurement of dietary carbohydrates. Food chemistry.

[b0375] Escalona V.H., Aguayo E., Gómez P., Artés F. (2004). Modified atmosphere packaging inhibits browning in fennel. LWT-Food Science and Technology.

[b0380] Fadel, M.A., Kurmestegy, L., Rashed, M., Rashed, Z., 2001. Date variety recognition and sugar content estimation via color analysis, The Second International Conference on Date Palms. Al-Ain, UAE.

[b0385] FAOSTAT, F., 2018. Crop statistics.

[b0390] Farber J., Harris L., Parish M., Beuchat L., Suslow T., Gorney J., Garrett E., Busta F. (2003). Microbiological safety of controlled and modified atmosphere packaging of fresh and fresh-cut produce. Comprehensive reviews in food science and food safety.

[b0395] Finkelman S., Navarro S., Rindner M., Dias R. (2006). Use of heat for disinfestation and control of insects in dates: laboratory and field trials. Phytoparasitica.

[b0400] Gebhardt S.E., Thomas R.G. (2002). Nutritive value of foods. US Government Printing. Office..

[b0405] Gherbawy Y.A., Elhariry H.M., Bahobial A.A.S. (2012). Mycobiota and mycotoxins (aflatoxins and ochratoxin) associated with some Saudi date palm fruits. Foodborne pathogens and disease.

[b0410] Ghnimi S., Umer S., Karim A., Kamal-Eldin A. (2017). Date fruit (Phoenix dactylifera L.): An underutilized food seeking industrial valorization. NFS journal.

[b0415] Glasner B., Botes A., Zaid A. (2002). Date harvesting, packinghouse management and marketing aspects. Date Palm Cultivation.

[b0420] Golshan Tafti A., Solaimani Dahdivan N., Yasini Ardakani S. (2017). Physicochemical properties and applications of date seed and its oil. International Food Research Journal.

[b0425] Gros-Balthazard M., Hazzouri K.M., Flowers J.M. (2018). Genomic insights into date palm origins. Genes.

[b0430] Hamad I., AbdElgawad H., Al Jaouni S., Zinta G., Asard H., Hassan S., Hegab M., Hagagy N., Selim S. (2015). Metabolic analysis of various date palm fruit (Phoenix dactylifera L.) cultivars from Saudi Arabia to assess their nutritional quality. Molecules.

[b0435] Hamad S.H. (2008). Microbial spoilage of date Rutab collected from the markets of Al-Hofuf City in the Kingdom of Saudi Arabia. Journal of food protection.

[b0440] Hammouda, H.d., Chérif, J.K., Trabelsi-Ayadi, M., Baron, A., Guyot, S., 2013. Detailed polyphenol and tannin composition and its variability in Tunisian dates (Phoenix dactylifera L.) at different maturity stages. Journal of agricultural and food chemistry 61, 3252-3263. 10.1021/jf304614j 23374033

[b0445] He Q., Luo Y., Chen P. (2008). Elucidation of the mechanism of enzymatic browning inhibition by sodium chlorite. Food Chemistry.

[b0450] He X., Deng H., Hwang H.-M. (2019). The current application of nanotechnology in food and agriculture. Journal of food and drug analysis.

[b0455] Homayouni A., Azizi A., Keshtiban A.K., Amini A., Eslami A. (2015). Date canning: a new approach for the long time preservation of date. Journal of food science and technology.

[b0460] Hui Y.H. (2008). Handbook of fruits and fruit processing.

[b0465] Huntrods D. (2011). Date profile.

[b0470] Hussain M.I., Farooq M., Syed Q.A. (2020). Nutritional and biological characteristics of the date palm fruit (Phoenix dactylifera L.)–A review. Food. Bioscience.

[b0475] Ibrahim S., Rahma M. (2009). Isolation and indentification of fungi associated with date fruits (Phoenix dactylifera, Linn) sold at Bayero University, Kano, Nigeria. Bayero Journal of Pure and Applied Sciences.

[b0480] Izumi H. (1999). Electrolyzed water as a disinfectant for fresh-cut vegetables. Journal of Food Science.

[b0485] Jemni M., Chniti S., Harbaoui K., Ferchichi A., Artés F. (2016). Partial vacuum and active modified atmosphere packaging for keeping overall quality of dates. J New Sci Agric Biotechnol.

[b0490] Jemni M., Chniti S., Soliman S.S. (2019). Date (Phoenix dactylifera L.) Seed Oil.

[b0495] Jemni M., Gómez P.A., Souza M., Chaira N., Ferchichi A., Otón M., Artés F. (2014). Combined effect of UV-C, ozone and electrolyzed water for keeping overall quality of date palm. LWT-Food Science and Technology.

[b0500] Jemni M., Otón M., Ramirez J.G., Artés-Hernández F., Chaira N., Ferchichi A., Artés F. (2014). Conventional and emergent sanitizers decreased Ectomyelois ceratoniae infestation and maintained quality of date palm after shelf-life. Postharvest biology and technology.

[b0505] Jemni M., Otón M., Souza M., Dhouibi M., Ferchichi A., Artés F. (2015). Ozone gas greatly reduced the survival of carob moth larvae in stored date palm fruit. Journal of New Sciences.

[b0510] Jemni M., Ramírez J., Otón M., Artés-Hernández F., Harbaoui K., Namsi A., Ferchichi A., Artés F. (2016). Passive modified atmosphere packaging and chilling storage for keeping overall quality of dates. VIII International Postharvest Symposium: Enhancing Supply Chain and Consumer Benefits-Ethical and Technological Issues.

[b0515] Jemni M., Ramirez J.G., Oton M., Artés-Hernandez F., Harbaoui K., Namsi A., Ferchichi A., Artés F. (2019). Chilling and Freezing Storage for Keeping Overall Quality of “Deglet Nour” Dates. Journal of Agricultural Science and Technology.

[b0520] Juhaimi F.A., Ghafoor K., Özcan M.M. (2012). Physical and chemical properties, antioxidant activity, total phenol and mineral profile of seeds of seven different date fruit (Phoenix dactylifera L.) varieties. International journal of food sciences and nutrition.

[b0525] Julia V., Macia L., Dombrowicz D. (2015). The impact of diet on asthma and allergic diseases. Nature Reviews Immunology.

[b0530] Kader, Awad, M.H., 2009. Harvesting and postharvest handling of dates. ICARDA, Aleppo, Syria 4, 15.

[b0535] Kader A. (2003). A perspective on postharvest horticulture (1978–2003). HortScience.

[b0540] Kader, A., 2007. Recommendations for maintaining postharvest quality. Department of Plant Science, University of California, Davis.

[b0545] Kader A., Kasmire R. (1984). Effects of ethylene on commodities during postharvest handling. Outlook.

[b0550] Kader A., Yahia E. (2011). Postharvest Biology and Technology of Tropical and Subtropical Fruits.

[b0555] Kahramanoglu I., Usanmaz S. (2019). Preharvest and postharvest treatments for increasing the rate of ripening of date palm fruit (Phoenix dactylifera L.) cv. Medjool. Progress in nutrition.

[b0560] Kchaou W., Abbès F., Blecker C., Attia H., Besbes S. (2013). Effects of extraction solvents on phenolic contents and antioxidant activities of Tunisian date varieties (Phoenix dactylifera L.). Industrial crops and products.

[b0565] Khalid S., Khalid N., Khan R.S., Ahmed H., Ahmad A. (2017). A review on chemistry and pharmacology of Ajwa date fruit and pit. Trends in food science & technology.

[b0570] Kim G.H., Kim J.E., Rhie S.J., Yoon S. (2015). The role of oxidative stress in neurodegenerative diseases. Experimental neurobiology.

[b0575] Kohli, S.K., Khanna, K., Bhardwaj, R., Abd_Allah, E.F., Ahmad, P., Corpas, F.J., 2019. Assessment of subcellular ROS and NO metabolism in higher plants: multifunctional signaling molecules. Antioxidants 8, 641. 10.3390/antiox8120641 PMC6943533 31842380

[b0580] Kondo N. (2010). Automation on fruit and vegetable grading system and food traceability. Trends in Food Science & Technology.

[b0585] Kumar S., Mukherjee A., Dutta J. (2020). Chitosan based nanocomposite films and coatings: Emerging antimicrobial food packaging alternatives. Trends in Food Science & Technology.

[b0590] Lallouche A., Kolodyaznaya V., Boulkrane M.S., Baranenko D. (2017). Low Temperature Refrigeration as an Alternative Anti-Pest Treatment of Dates. Environmental & Climate Technologies.

[b0595] Lee D.-J., Schoenberger R., Archibald J., McCollum S. (2008). Development of a machine vision system for automatic date grading using digital reflective near-infrared imaging. Journal of food Engineering.

[b0600] Lobo M.G., Yahia E.M., Kader A.A., Siddiq M., Aleid S.M., Kader A.A. (2014). Biology and Postharvest Physiology of Date Fruit. Dates: Postharvest science, processing technology and health benefits.

[bib881] Lyddy R (2009). Chapter 36 - Nanotechnology. Information Resources in Toxicology (Fourth Edition).

[b0605] Mansouri A., Embarek G., Kokkalou E., Kefalas P. (2005). Phenolic profile and antioxidant activity of the Algerian ripe date palm fruit (Phoenix dactylifera). Food chemistry.

[b0610] Mard S.A., Jalalvand K., Jafarinejad M., Balochi H., Naseri M.K.G. (2010). Evaluation of the antidiabetic and antilipaemic activities of the hydroalcoholic extract of Phoenix dactylifera palm leaves and its fractions in alloxan-induced diabetic rats. The Malaysian journal of medical sciences: MJMS.

[b0615] Marouf A., Amir-Maafi M., Shayesteh N. (2013). Two-sex life table analysis of population characteristics of almond moth, Cadra cautella (Lepidoptera: Pyralidae) on dry and semi-dry date palm varieties. Journal of Crop Protection.

[bib883] Martins S.I.F.S, Jongen W.M.F, van Boekel M.A.J.S (2000). A review of Maillard reaction in food and implications to kinetic modelling. Trends in food science & technology.

[b0620] Martín-Sánchez A.M., Cherif S., Ben-Abda J., Barber-Vallés X., Pérez-Álvarez J.Á., Sayas-Barberá E. (2014). Phytochemicals in date co-products and their antioxidant activity. Food chemistry.

[b0625] Martínez-Hernández G.B., Artés-Hernández F., Gómez P.A., Formica A.C., Artés F. (2013). Combination of electrolysed water, UV-C and superatmospheric O2 packaging for improving fresh-cut broccoli quality. Postharvest Biology and Technology.

[b0630] Masmoudi M., Besbes S., Blecker C., Attia H. (2010). Preparation and characterization of jellies with reduced sugar content from date (Phoenix dactylifera L.) and lemon (Citrus limon L.) by-products. Fruits.

[b0635] Mediouni J., Fuková I., Frydrychová R., Dhouibi M.H., Marec F. (2004). Karyotype, sex chromatin and sex chromosome differentiation in the carob moth, Ectomyelois ceratoniae (Lepidoptera: Pyralidae). Caryologia.

[b0640] Mohamed Lemine, F.M., Mohamed Ahmed, M.V.O., Ben Mohamed Maoulainine, L., Bouna, Z.e.A.O., Samb, A., O. Boukhary, A.O.M.S., 2014. Antioxidant activity of various Mauritanian date palm (P hoenix dactylifera L.) fruits at two edible ripening stages. Food science & nutrition 2, 700-705. 10.1002/fsn3.167 PMC4256575 25493188

[b0645] Mohandass S., Arthur F., Zhu K., Throne J.E. (2007). Biology and management of Plodia interpunctella (Lepidoptera: Pyralidae) in stored products. Journal of Stored Products Research.

[b0650] Monzon M., Biasi B., Simpson T., Johnson J., Feng X., Slaughter D., Mitcham E. (2006). Effect of radio frequency heating as a potential quarantine treatment on the quality of ‘Bing’sweet cherry fruit and mortality of codling moth larvae. Postharvest biology and technology.

[b0655] Moss J.W., Ramji D.P. (2016). Nutraceutical therapies for atherosclerosis. Nature Reviews Cardiology.

[b0660] Mrabet A., Jiménez-Araujo A., Fernández-Bolaños J., Rubio-Senent F., Lama-Muñoz A., Sindic M., Rodríguez-Gutiérrez G. (2016). Antioxidant phenolic extracts obtained from secondary Tunisian date varieties (Phoenix dactylifera L.) by hydrothermal treatments. Food Chemistry.

[b0665] Mrabet A., Rodríguez-Gutiérrez G., Rubio-Senent F., Hamza H., Rodríguez-Arcos R., Guillén-Bejarano R., Sindic M., Jiménez-Araujo A. (2017). Enzymatic conversion of date fruit fiber concentrates into a new product enriched in antioxidant soluble fiber. LWT.

[b0670] Munier, P., 1973. Le palmier-dattier. Maisonneuve & Larose.

[b0675] Mustafa A.B., Harper D.B., Johnston D.E. (1986). Biochemical changes during ripening of some Sudanese date varieties. Journal of the Science of Food and Agriculture.

[b0680] Nasser L. (2017). Fungal Contamination and Invertase Activity in Dates and Date Products in Saudi Arabia. American Journal of Food Technology.

[b0685] Navarro S. (2006). Postharvest treatment of dates. Stewart Postharvest Review.

[b0690] Nehdi I.A., Sbihi H.M., Tan C.P., Rashid U., Al-Resayes S.I. (2018). Chemical composition of date palm (Phoenix dactylifera L.) seed oil from six Saudi Arabian cultivars. Journal of food science.

[b0695] Niakousari M., Erjaee Z., Javadian S. (2010). Fumigation characteristics of ozone in postharvest treatment of kabkab dates (Phoenix dactylifera L.) against selected insect infestation. Journal of food protection.

[b0700] Njoroge, J.B., Ninomiya, K., Kondo, N., Toita, H., 2002. Automated fruit grading system using image processing, Proceedings of the 41st SICE Annual Conference. SICE 2002. IEEE, pp. 1346-1351.

[b0705] Omamor I.B., Hamza A. (2006). The effects of relative humidity and temperature on disease development in stored date fruits, III International Date Palm Conference 736. Acta Horticulturae.

[b0710] Ooi, P.A.C., Winotai, A., Peña, J.E., 2002. Pests of minor tropical fruits, In: Peña, J.E., Sharp, J.L., Wysoki, M. (Eds.), Tropical fruit pests and pollinators: biology, economic importance, natural enemies and control. CABI, pp. 315-330.

[b0715] Palou L., Rosales R., Taberner V., Vilella-Espla J. (2016). Incidence and etiology of postharvest diseases of fresh fruit of date palm (Phoenix dactylifera L.) in the grove of Elx (Spain). Phytopathologia Mediterranea.

[b0720] Phulpoto N.N., Shah A.B., Shaikh F.M. (2012). Challenges faced by rural women in dates processing industry in Khairpur Mirs. Australian Journal of Business and Management Research.

[b0725] Pompella A., Sies H., Wacker R., Brouns F., Grune T., Biesalski H.K., Frank J. (2014). The use of total antioxidant capacity as surrogate marker for food quality and its effect on health is to be discouraged. Nutrition.

[b0730] Popenoe P.B. (1913). Date growing in the Old and New Worlds.

[b0735] Pourdarbani R., Ghassemzadeh H.R., Seyedarabi H., Nahandi F.Z., Vahed M.M. (2015). Study on an automatic sorting system for Date fruits. Journal of the Saudi Society of Agricultural Sciences.

[b0740] Prosky L., Asp N.-G., Schweizer T.F., Devries J.W., Furda I. (1988). Determination of insoluble, soluble, and total dietary fiber in foods and food products: interlaboratory study. Journal of the Association of Official Analytical Chemists.

[b0745] Quaglia M., Santinelli M., Sulyok M., Onofri A., Covarelli L., Beccari G. (2020). Aspergillus, Penicillium and Cladosporium species associated with dried date fruits collected in the Perugia (Umbria, Central Italy) market. International journal of food microbiology.

[b0750] Rafaeli A., Kostukovsky M., Carmeli D. (2006). Successful disinfestations of sap-beetle contaminations from organically grown dates using heat treatment: A case study. Phytoparasitica.

[b0755] Ragab W., Ramadan B., Abdel-Sater M. (2001). Mycoflora and aflatoxins associated with saidy date affected by technological processes. The Second International Conference on Date Palms.

[b0760] Rico D., Martín-Diana A.B., Barry-Ryan C., Frías J.M., Henehan G.T., Barat J.M. (2008). Use of neutral electrolysed water (EW) for quality maintenance and shelf-life extension of minimally processed lettuce. Innovative food science & emerging technologies.

[b0765] Rose D.C., Chilvers J. (2018). Agriculture 4.0: Broadening responsible innovation in an era of smart farming. Frontiers in Sustainable Food Systems.

[b0770] Rygg G. (1975). Date Development, Handling and Packing in the US. USDA Agric. Handbook.

[b0775] Saafi, E.B., El Arem, A., Mahmoud, O., Ferchichi, A., Hammami, M., Achour, L., 2009. Date palm fruit in Tunisia: Chemical screening and analysis of phenolic acids and carotenoids by Thin-Layer Chromatography, Revue des Régions Arides–Numéro spécial–24 (2/2010) Actes du 3ème Meeting International “Aridoculture et Cultures Oasisennes: Gestion et Valorisation des Ressources et Applications Biotechnologiques dans les Agrosystèmes Arides et Sahariens” Jerba (Tunisia), pp. 15-17.

[b0780] Salehi, B., Ata, A., V Anil Kumar, N., Sharopov, F., Ramírez-Alarcón, K., Ruiz-Ortega, A., Abdulmajid Ayatollahi, S., Valere Tsouh Fokou, P., Kobarfard, F., Amiruddin Zakaria, Z., 2019. Antidiabetic potential of medicinal plants and their active components. Biomolecules 9, 551. 10.3390/biom9100551 PMC6843349 31575072

[b0785] Sandhya, 2010. Modified atmosphere packaging of fresh produce: Current status and future needs. LWT-Food Science and Technology 43, 381-392.

[b0790] Saqib S., Zaman W., Ayaz A., Habib S., Bahadur S., Hussain S., Muhammad S., Ullah F. (2020). Postharvest disease inhibition in fruit by synthesis and characterization of chitosan iron oxide nanoparticles. Biocatalysis and Agricultural Biotechnology.

[b0795] Shaghaghian S., Niakousari M., Javadian S. (2014). Application of ozone post-harvest treatment on Kabkab date fruits: effect on mortality rate of Indian meal moth and nutrition components. Ozone: Science & Engineering.

[b0800] Shenasi M., Aidoo K.E., Candlish A.A. (2002). Microflora of date fruits and production of aflatoxins at various stages of maturation. International Journal of Food Microbiology.

[b0805] Shenasi M., Candlish A.A.G., Aidoo K.E. (2002). The production of aflatoxins in fresh date fruits and under simulated storage conditions. Journal of the Science of Food and Agriculture.

[b0810] Siddiq, M., Greiby, I., 2014. Overview of date fruit production, postharvest handling, processing, and nutrition, In: M. Siddiq, S.A., and AA Kader. John Wiley and Sons, Chichester, United Kingdom (Ed.), Dates: postharvest science, processing technology and health benefits, 1st edition ed. John Wiley and Sons, Chichester, United Kingdom, pp. 1-28.

[b0815] Tavakolian M.S., Silaghi F.A., Fabbri A., Molari G., Giunchi A., Guarnieri A. (2013). Differentiation of post harvest date fruit varieties non-destructively using FT-NIR spectroscopy. International journal of food science & technology.

[b0820] Tavakolipour H., Kalbasi-ashtari A. (2007). Influence of gums on dough properties and flat bread quality of two Persian wheat varieties. Journal of food process engineering.

[b0825] Toivonen P.M., Brandenburg J.S., Luo Y. (2009). Modified atmosphere packaging for fresh-cut produce. Modified and controlled atmospheres for the storage, transportation, and packaging of horticultural commodities.

[b0830] Tomás-Callejas A., Martínez-Hernández G., Artés F., Artés-Hernández F. (2011). Neutral and acidic electrolyzed water as emergent sanitizers for fresh-cut mizuna baby leaves. Postharvest Biology and Technology.

[b0835] USDA, 2020. Food Data Central: Dates, medjool.

[b0840] Wakil W., Faleiro J.R., Miller T.A. (2015). Sustainable pest management in date palm: Current status and emerging challenges.

[b0845] Walsh K.B., Blasco J., Zude-Sasse M., Sun X. (2020). Visible-NIR ‘point’spectroscopy in postharvest fruit and vegetable assessment: The science behind three decades of commercial use. Postharvest Biology and Technology.

[b0850] Wang C.Y. (1989). Chilling injury of fruits and vegetables. Food reviews international.

[b0855] Warner R., Barnes M., Laird E. (1990). Chemical control of a carob moth, Ectomyelois ceratoniae (Lepidoptera: Pyralidae), and various nitidulid beetles (Coleoptera) on ‘Deglet Noor’dates in California. Journal of Economic Entomology.

[b0860] Yahia E.M. (2011). Postharvest biology and technology of tropical and subtropical fruits: fundamental issues.

[b0865] Yahia E.M., Kader A. (2011). Date (Phoenix dactylifera L.), Postharvest biology and technology of tropical and subtropical fruits.

[b0870] Yahia, E.M., Lobo, M.G., kader, A.A., 2014. Harvesting and Postharvest Technology of Dates, In: Siddiq, M., Aleid, S.M., Kader, A.A. (Eds.), Dates: Postharvest science, processing technology and health benefits. John Wiley & Sons Ltd, pp. 105-135.

[b0875] Zaid A., De Wet P., Djerbi M., Oihabi A. (1999). Chapter XII diseases and pests of date palm. FAO Plant Production and Protection Papers.

[b0880] Zouba A., Khoualdia O., Diaferia A., Rosito V., Bouabidi H., Chermiti B. (2009). Microwave treatment for postharvest control of the date moth Ectomyelois ceratoniae. Tunisian Journal of Plant Protection.

